# Characterizing Attention Resource Capacity in Autism: A Multiple Object Tracking Study

**DOI:** 10.1007/s10803-023-05974-z

**Published:** 2023-06-21

**Authors:** Domenico Tullo, Bianca Levy, Jocelyn Faubert, Armando Bertone

**Affiliations:** 1 University of California Irvine, Irvine, CA USA; 2 McGill University, Montréal, Québec Canada; 3 Université de Montréal, Montréal, Québec Canada

**Keywords:** Attention, Autism, Attention resource capacity, multiple object tracking, Fluid reasoning intelligence

## Abstract

The extant literature aimed at characterizing attentional capability in autistics has presented inconsistent findings. This inconsistency and uncertainty may be the product of different theoretical and methodological approaches used to define attention in autism. In the current study, we investigate whether the allocation of attentional resources to task demands, and attention resource capacity, differs between autistics with no comorbid attention-deficit diagnosis (n = 55) and age-matched neurotypicals (n = 55). We compared differences in capacity and the allocation of resources by manipulating attentional load in a Multiple Object-Tracking (MOT) task, a robust, versatile, and ecological measure of selective, sustained, and distributed attention. While autistics demonstrated lower MOT performance, this difference disappeared when we accounted for fluid reasoning intelligence. Additionally, the similarity in the trend of MOT performance at increasing levels of attentional load between autistics and neurotypicals suggests no differences in the allocation of attentional resources to task demands. Taken together, our study suggests that higher-order cognitive abilities, such as intelligence, should be considered when characterizing attention across the autistic population in research. Similarly, our findings highlight the importance of considering cognitive competence when assessing attentional capabilities in autistic individuals, which could have significant implications for clinical diagnosis, treatment, and support.

## Introduction

Autism spectrum disorder (ASD) is a heterogeneous neurodevelopmental condition defined by atypical social communication and social interactions across multiple contexts, and by restricted, repetitive patterns of behavior, interests, or activities (American Psychiatric Association 2013). While deficits in attention are not considered a core diagnostic criterion of ASD, a significant proportion of autistic^1^ youth present attention-related symptoms that are consistent with attention-deficit/hyperactivity disorder (ADHD; Davis and Kollins 2012; Leitner 2014; Sikora et al. 2012). In fact, the number of clinical studies characterizing attention in ASD and those investigating the co-occurrence of ASD and ADHD has accelerated since the latest iteration of the Diagnostic and Statistical Manual of Mental Disorders (Antshel & Russo, 2019; Burack et al., 2016; Dellapiazza et al., 2018), which recognizes a concurrent ASD and ADHD diagnosis (American Psychiatric Association 2013).

Most of what is known about attention and ASD originates from studies assessing ‘high-functioning’ autistics using tasks and measures that are not necessarily adapted to either the participants’ cognitive capability (i.e., intellectual functioning) or cognitive style (i.e., non-verbal or verbal; Joseph et al. 2002). In addition, the inconsistent findings in research characterizing attentional abilities in autism are also related to the difficulty in operationally defining attention (see Ames and Fletcher-Watson 2010; Burack et al. 2016 for reviews). This inconsistency is reflected pragmatically by the plethora of experimental tasks and approaches used, which have resulted in findings that suggest intact (Lopez et al., 2005; Ozonoff & Strayer, 1997), decreased (Burack, 1994; Occelli et al., 2013; Renner et al., 2006) and even superior attentional capabilities in autistics compared to neurotypicals (Collignon et al., 2013; Joseph et al., 2009; Kaldy et al., 2016).

Additionally, the majority of studies attempting to characterize attention in autism have targeted specific sub-components of attention (e.g., selective attention; Dellapiazza et al. 2018). For example, previous research has shown that autistic persons have the ability to selectively attend to increased levels of stimuli compared to neurotypicals (Adams & Jarrold, 2012; Remington et al., 2009, 2012). According to perceptual load theory, the effectiveness of selective attention is contingent upon the level of perceptual load of a given task, stimulus, or environment (Lavie, 1995, 2005). At a low perceptual load, leftover processing capacity shifts to the perception of irrelevant stimuli when allocating attention to task-relevant stimuli at low perceptual load; whereas, at a high perceptual load, processing capacity is reached and task-irrelevant stimuli are ignored (Lavie, 1995). Previous work has suggested that autistic persons may have higher perceptual capacities than neurotypicals; however, this means they are more susceptible to processing distracting information (Adams & Jarrold, 2012). Although such measures and approaches are empirically valid, a more eclectic and ecologically valid characterization of attentional capability is ideal (Powell et al., 2017; Scerif, 2010). For instance, the ability to selectively assign, sustain, and distribute attention across relevant information while ignoring irrelevant information is a robust indicator of functional attention used to navigate the everyday environment and a core component of learning capability (Scholl, 2009).

Completing real-world activities such as finding Waldo (Ennesser & Medioni, 1995), playing an action-based video game (Green & Bavelier, 2003), or focusing on relevant classroom-based material while ignoring distractions (Steinmayr et al., 2010) requires the allocation of attentional resources that draw from a limited capacity. Therefore, attention resource capacity, or the limited reserve of readily available attentional resources that can be allocated to the demands of an attention-based task (Alvarez et al., 2005; Alvarez & Cavanagh, 2004; Alvarez & Franconeri, 2007), can be used as an accurate and descriptive measure of attentional capability (Tullo, Faubert, et al. 2018). Individual differences in attention resource capacity can be assessed by measuring performance on a task targeting attention while manipulating that same task’s attentional load (Alnæs et al., 2014; Alvarez & Franconeri, 2007; Tombu & Seiffert, 2008; Tullo et al., 2020; Tullo, Faubert, et al. 2018). As such, the Multiple Object Tracking (MOT) task is ideal for characterizing the allocation of attentional resources at different levels of attentional load (Tullo et al., 2020) by measuring MOT performance across conditions with increasing target items to be tracked (Alvarez & Franconeri, 2007).

The MOT task involves tracking a set of target objects that move among physically indistinguishable distractor objects for a short period of time (Pylyshyn & Storm, 1988). MOT paradigms have been used to characterize differences in tracking capability across developmental stages (Trick et al., 2005), and in atypically-developing populations (O’Hearn et al., 2010). A significant advantage of isolating attention with MOT within atypically developing populations is the task’s feasibility across a large age range and cognitive capability (Archambault et al., 2021; Tullo, Guy, et al. 2018). Findings from previous studies examining MOT capability across the autistic population are inconsistent and often interpreted as reflecting visuoperceptual ability (i.e., visual grouping; see Evers et al. 2014; Koldewyn et al. 2013 for examples; Van der Hallen et al. 2015) rather than attention (see Scholl 2009). However, the use of traditional methods of defining MOT capability, such as object limits and accuracy rates has produced inconsistent findings for studies assessing MOT capability in both typically and atypically developing populations (Tullo et al., 2020, 2023; Tullo, Faubert, et al. 2018).

MOT capability has been previously defined as a categorical object limit where performance is defined by the maximum number of target objects tracked (i.e., ability to track two vs. three target objects), which has contributed to the inconsistent findings in both autistic (Koldewyn et al., 2013) and neurotypically-developing persons (Fougnie & Marois, 2006; Viswanathan & Mingolla, 2002). In Tullo et al. (2018), the authors outlined the relationship between MOT capability and fluid reasoning intelligence. To do this, they used a continuous ratio variable, the average speed score, as a metric for MOT capability and attention resource capacity. The study reported that the average speed score, which represents the maximum speed at which participants successfully tracked all target objects, decreased as the number of target objects increased, consistent with previous research by Alvarez and Franconeri (2007) that used object velocity to describe the allocation of attentional resources to task demands. The results from Tullo et al. (2018) also revealed a positive association between fluid reasoning intelligence and MOT capability, and that performance on the MOT task differed between individuals with low and high fluid reasoning intelligence. As such, the authors argue that the use of the average speed score as a metric for MOT capability has advantages over using accuracy rates and/or object limits. Specifically, this metric provides a continuous measure of performance that can better account for individual differences. Similarly, accuracy rates are also limited by a narrow range of scores.

Here, we characterize MOT performance using a continuous outcome variable, defined by the maximum speed at which target objects can be tracked. This approach has been demonstrated to be the preferred approach given the accuracy in characterizing attentional capacity across diverse individuals (Alvarez & Franconeri, 2007; Tombu & Seiffert, 2008; Tullo et al., 2020; Tullo, Faubert, et al. 2018). MOT performance is used in the current study to assess attention resource capacity in a group of adolescent and adult autistics. We measure performance on the MOT task at increasing levels of attentional load, by increasing the number of target objects to be tracked (Alvarez & Franconeri, 2007; Meyerhoff et al., 2017; Scholl, 2009). Performance, defined as the maximum speed participants can successfully track all target items, is an optimal approximation of attention resource capacity (Chen et al., 2013; Holcombe & Chen, 2012, 2013; Tombu & Seiffert, 2008). In addition, we are also interested in assessing whether fluid reasoning (or non-verbal) intelligence could be a significant predictor of MOT capability in autism since a robust positive relationship was found in neurotypical adults (Tullo et al., 2020; Tullo, Faubert, et al. 2018). However, most studies examining the relationship between tracking capability and higher-order cognition in autistic populations have not considered fluid reasoning intelligence as a significant predictor and instead have used it as inclusionary and/or exclusionary criteria (Evers et al., 2014; Koldewyn et al., 2013).

In fact, previous research has demonstrated elevated fluid reasoning intelligence compared to verbal intelligence in the autistic population (Dawson et al., 2007; Nader et al., 2015). The present study extends this work by investigating the relationship between fluid reasoning intelligence and attention resource capacity in individuals with and without an ASD diagnosis. In addition, Tullo et al. (2018) reported that individuals with a fluid reasoning intellectual style, characterized by a bias in non-verbal intelligence scores compared to verbal intelligence scores, outperformed those with a verbal intellectual style, characterized by a bias in verbal IQ scores over non-verbal IQ scores, on the MOT task. This effect was particularly robust in high-load conditions. The present study aims to investigate whether this relationship extends to the autistic population, given the elevated levels of fluid reasoning capability over verbal capability (Dawson et al., 2007; Nader et al., 2015), and whether it can account for differences in attention resource capacity between individuals with and without autism.

The aim of the current study is to investigate whether attention resource capacity, the limited reserve of attentional resources, differs between individuals with autism and age-matched neurotypicals. The concept of attention resource capacity has been studied extensively in the literature, and it has been found that it plays a critical role in attentional functioning and in the performance of tasks that require attentional processing (Chen et al., 2013; Holcombe & Chen, 2012; Tombu & Seiffert, 2008). Given previous findings that suggest a strong link between fluid reasoning intelligence and MOT performance (Tullo et al., 2020, 2023; Tullo, Faubert, et al. 2018), we are particularly interested in exploring the relationship between fluid reasoning intelligence and attention resource capacity in autism. Fluid reasoning, a cognitive construct that refers to the ability to reason and solve problems in novel situations, is considered a key component in academic skills such as math (see Clark et al. 2021 for example) and general intelligence (e.g., Buehner et al. 2006). Recent research has shown that fluid reasoning is positively associated with performance on MOT tasks (see Tullo et al. 2020, 2023; Tullo, Faubert, et al. 2018), which suggests that individuals with higher fluid reasoning scores may have more attentional resources available for the task. Therefore, the aim of the current study is to explore whether autistics present different attention resource capacities compared to neurotypicals and if this difference can be explained fluid reasoning intelligence.

## Method

### Participants

#### Autistic Group

Forty (40) males and fifteen (15) female participants with an autism spectrum disorder (ASD) diagnosis (n = 55) were recruited through the Clinique d’évaluation des troubles envahissants du développement (CETED) at Rivière-des-Prairies Hospital, and the Summit Center for Education, Research, and Training (SCERT), both located in Montreal, QC, Canada. Participants ranged in age from 12 to 30 years (M = 18.72, SD = 4.34). Individuals were excluded from participating if they (i) were taking medication that would affect their attention, (ii) had a comorbid diagnosis of ADHD, (iii) had a personal or family history of a seizure disorder (e.g., epilepsy), or (iv) any condition that would affect their vision. All autistic participants met diagnostic criteria for ASD with a diagnosis confirmed by a psychologist or psychiatrist using systematic observation and standardized assessment including the Autism Diagnosis Observation Schedule (ADOS; Lord et al. 2000), or a combination of the ADOS and the Autism Diagnosis Interview-Revised (ADI-R; Lord et al. 1994). Autism community members were not directly involved in the study.

#### Neurotypical (NT) Group

Twenty-six (26) male and twenty-nine (29) female neurotypical participants (n = 55) were recruited via online advertisements and tested at McGill University in Montreal, QC, Canada. Participants ranged in age from 12 to 30 years (M = 18.93, SD = 4.50). Individuals were excluded from participating in the study if they (i) were taking medication that would affect their attention (i.e., stimulant, or sedative medication), (ii) had a diagnosis of ADHD and/or ASD (iii) had a personal or family history of a seizure disorder (e.g., epilepsy), or (iv) any condition that would affect their vision.

#### Demographic Variables

As depicted in Tables 1, autism and NT groups were matched on chronological age; *t*(108) = -0.26, *p* = .798, Cohen’s *d* = -0.05, 95% Confidence Interval (CI) [-0.53, 0.43], and performance on a separate, clinically validated measure of attention, as measured by a *d*’ t-score of the Conners Continuous Performance Test - Third Edition, (CPT-3; Conners 2014); *t*(108) = -0.36, *p* = .721, *d* = -0.07, 95% CI [-0.44, 0.31]). There was a discrepancy in intelligence where the NT group had a significantly greater mean Full Scale Wechsler-defined IQ (FSIQ); *t*(108) = -4.83, *p* < .001, *d* = -0.93, 95% CI [-1.31, -0.53], perceptual reasoning IQ (PRI); *t*(108) = -3.68, *p* < .001, *d* = -0.71, 95% CI [-1.09, -0.31], and verbal IQ (VCI); *t*(108) = -4.34, *p* < .001, *d* = -0.84, 95% CI [-1.22, -0.44] scores than the autism group. The autism group also had a larger range (i.e., lower minimum score) for FSIQ, VCI, and PRI scores compared to the NT group. Specific data on race/ethnicity and socioeconomic status were not collected or recorded.

**Table 1:** *Participant age, IQ, and performance on a measure of attention by group*

	**Autism (40 M, 15 F)**				**Neurotypical (26 M, 29 F)**			
	**Mean**	**SD**	**Range**		**Mean**	**SD**	**Range**	***p***
**Age (years** **)**	18.72	4.37	12–30		18.93	4.50	12–30	0.798
**FSIQ**	89.82	20.41	59–137		105.67	13.23	76–136	< 0.001
**PRI**	93.30	21.55	58–133		106.20	14.26	70–137	< 0.001
**VCI**	88.37	21.55	55–147		103.47	13.86	70–131	< 0.001
**CPT-3** ***d*’**	48.56	11.54	25–75		49.29	9.65	19–68	0.721

***Note.*** Standard scores reported for the Wechsler defined IQ scores by Full-Scale Intelligence Quotient (FSIQ), Verbal Comprehension Index (VCI) and Perceptual Reasoning Index (PRI). CPT-3 performance represented by t-scores on d’ (i.e., the primary outcome variable)

### Measures

#### Multiple Object Tracking (MOT)

**Figure 1:**
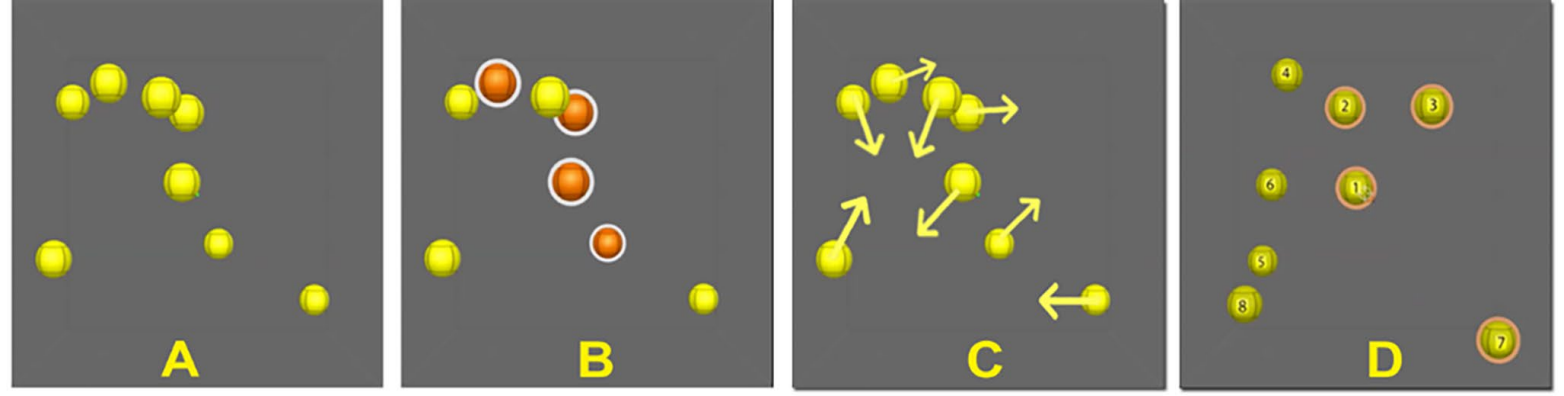
Illustration of an MOT trial comprised of four segments. (A) All 8 objects (i.e., spheres) are displayed in the visual field. (B) The target objects (i.e., trial with four targets) are highlighted orange. (C) Target objects change back to yellow, and all objects move randomly throughout the visual field. (D) Identifying numbers (i.e., 1-8) appear on the objects

A single MOT trial is broken down into four segments (see Fig. 1). Trials began with a presentation of eight yellow objects (i.e., spheres) randomly fixed across a 3D virtual cubic space (Fig. 1A). Next, objects changed from yellow to orange for one second, these represented the objects the participant was asked to track (i.e., target objects). Depending on the condition of attentional load, either one, two, three, or four objects were highlighted in orange. Figure 1B illustrates an example of a trial with four targets. The target objects returned to their original colour (i.e., yellow) and all objects moved randomly throughout the 3D space (Fig. 1C). After eight seconds of movement, the objects stopped moving and an identifying number from one to eight appeared on each object. Participants then verbally identified the object(s) that they believed to be target object(s). Their answers were entered manually by the experimenter on the computer’s numerical keypad. A yellow halo appeared on all selected objects and the participant confirmed their responses (Fig. 1D). The proceeding trial began after the selected objects were finalized. The MOT task was presented to the participant in three dimensions using a Sony HMZ-T1 wearable head-mounted display (HMD).

#### Wechsler Abbreviated Scale of Intelligence - Second Edition

. All autistic and neurotypical participants completed the Wechsler Abbreviated Scale of Intelligence, Second Edition (WASI-II; Wechsler 2011). The WASI-II is a brief measure of cognitive ability for individuals aged 6 to 90 years. It includes four subtests that factor into verbal comprehension (VCI) and perceptual reasoning (PRI) indices, as well as a full-scale intelligence quotient (FSIQ). Six (6) autistic participants, recruited from the shared CETED database at Rivière-des-Prairies Hospital, already had valid Wechsler IQ scores at the time of testing in the present study. These were obtained during their recent participation in other, unrelated research studies. Thus, VCI, PRI, and FSIQ scores from related adult and child comprehensive Weschler intelligence scales were used for those participants in place of WASI-II scores. These tests included the Wechsler Adult Intelligence Scale, 4th Edition (WAIS-IV), and the Wechsler Intelligence Scale for Children, 4th Edition (WISC-IV).

#### Conners Continuous Performance Test - Third Edition

. All participants completed the Conners Continuous Performance Test - Third Edition (CPT-3; Conners 2014), a 14 fourteen-minute-long computerized and clinically validated measure of attention. Participants are asked to respond to letters flashed on the screen (by pressing the spacebar) as quickly and accurately as possible, but to inhibit their responses when for the letter “X” only. The detectability (d’) t-score, defined as the ability to distinguish between targets and non-targets, was used as the primary outcome measure of attention. More specifically, the measure characterizes inattention, impulsivity, and sustained attention. The clinical cut-off for suggesting the presence of attentional problems with the CPT-3 is at a *t*-score of 60.

### Procedure

The WASI-II intelligence test, the CPT-3, and the MOT tasks were administered in a random order to control for any effects of fatigue. To ensure participants comprehended the objective of the MOT task, qualifying trials were administered before testing began. Specifically, participants needed to successfully track one out of eight objects in the first two of three trials. If unsuccessful, the instructions were re-explained to the participant, and they had to obtain two correct responses on the next three trials to qualify for testing. The research assistant controlled the progression throughout the task by entering the participants’ verbal responses manually on the laptop running the MOT task. All participants qualified for the study.

Participants had to track object set sizes of one, two, three, and four target objects over two blocks. Two blocks of trials for each level of attentional load (i.e., target set size) were presented in random order to each participant, with the number of trials in each level of the attentional load depending on the participant’s performance. The speed of the objects increased or decreased in subsequent trials depending on whether participants identified all target objects correctly in the previous trial (i.e., one up/one down staircase procedure; see Kaernbach 1991; Levitt 2005). Initial object speed was set at 68 cm/second displacement, and speed increased or decreased by 0.05 log. Object speeds ranged from 0.68 cm/second to 544 cm/second. The MOT task ended once six inversions occurred where an inversion is defined as a correct answer followed by an incorrect answer, and vice-versa. The geometric mean of speed comprised of the speed at each of the six inversions was calculated for each block of trials, and the average of the two blocks’ speed scores was used as the outcome measure of MOT performance at that condition of attentional load (Tullo, Faubert, et al. 2018).

### Design and Analyses

We conducted a two-step hierarchical multiple regression analysis to examine differences in MOT performance between the autism and NT groups. Step 1 included group and attentional load (i.e., at one, two, three, and four target objects) as predictors of MOT performance (i.e., average speed score). Planned *t*-tests were conducted at each level of attentional load between NT and autism groups. Additionally, we borrowed methodology from similar research in the neurotypical adult population (Tullo et al., 2020; Tullo, Faubert, et al. 2018) to characterize the trends in MOT performance and the allocation of attentional resources by autism and NT groups. First, we fit MOT performance to a decreasing logarithmic function to characterize MOT capability between groups. Second, we tested linearity between the log of performance (i.e., average speed score) and attentional load (i.e., log-log plot) to describe the allocation of attentional resources to task demands.

In Step 2 of the hierarchical regression, we added CPT-3 performance (*d’ t*-score) and WASI-II intelligence subscale scores of verbal (i.e., VCI score) and fluid reasoning (i.e., PRI score) intelligence. Here, we evaluated their unique contribution to explaining individual differences in tracking capability beyond group differences (i.e., Autism and NT). Lastly, we further explored whether MOT performance differed between verbal and fluid-reasoning intellectual styles as a function of NT and Autism groups using three-way mixed-design ANOVA with group and style as between-subjects factors and condition of load as the within-subjects factor.

## Results

### Attentional Load

The first step in the hierarchical multiple regression model yielded a significant model with attentional load (i.e., tracking one through four objects) and group (i.e., autism as the reference category), and the interaction between attentional load and group: *F*(3, 432) = 299.8, *p* < .001, *R*^2^ = 0.68, *Adj. R*^2^ = 0.67. While the interaction between the level of attentional load and group: *b =* 1.57, *t*(432) = 0.41, *p* = .684, 95% CI [-5.99, 9.12] was not a statistically significant predictor of performance, attentional load (*b =* -57.80, *t*(432) = -21.37, *p* < .001, 95% CI [-63.12, -52.49]) and group (*b =* -22.26, *t*(432) = -2.12, *p* = .035, 95% CI [-42.94, -1.57]) were statistically significant predictors of MOT capability (see Table 2). These results suggest that MOT capability decreased as the number of target objects increased from one to four. In addition, when collapsed across conditions of attentional load, the NT group could track target objects at faster speeds than the autism group.

**Table 2:** *Stepwise regression examining the predictive validity of MOT capability*

**Step 1**						
**Predictor**	***b***	***b*** ** 95%** ** CI**	***s*** **r** ^***2***^	***s*r**^***2***^ ** 95%** ** CI**	***p***	**Fit**
**(Intercept)**	290.81	[276.25, 305.37]			< 0.001	
**Condition**	-57.80	[-63.12, -52.49]	0.34	[0.28, 0.41]	< 0.001	
**ASD Dx**	-22.26	[-42.94, -1.57]	0.00	[-0.00, 0.01]	0.035	
**Condition by Dx**	1.56	[-5.99, 9.12]	0.00	[-0.00, 0.00]	0.648	
						*R*^*2*^ = 0.676, *R*^*2*^_*(ADJ)*_ *= 0.673, p < .001*
						95% CI [0.63,0.71]
**Step 2**						
**Predictor**	***b***	***b*** ** 95%** ** CI**	***s*** **r** ^***2***^	***s*r**^***2***^ ** 95%** ** CI**	***p***	**Fit**
**(Intercept)**	222.91	[180.38, 265.44]			< 0.001	
**Condition**	-57.80	[-62.72, -52.88]	0.34	[0.28, 0.40]	< 0.001	
**ASD Dx**	-12.61	[-32.15, 6.92]	0.00	[-0.00, 0.00]	0.205	
**CPT-3** ***d*** **’** **t-** **score**	-0.33	[-0.73, 0.08]	0.00	[-0.00, 0.01]	0.112	
**PRI**	0.94	[0.66, 1.21]	0.03	[0.01, 0.05]	< 0.001	
**VCI**	-0.15	[-0.42, 0.12]	0.00	[-0.00, 0.00]	0.267	
**Condition by Dx**	1.56	[-5.43, 8.56]	0.00	[-0.00, 0.00]	0.660	
						*R*^*2*^ = 0.724, *R*^*2*^_*(ADJ*_*= 0.72, p < .001*
						95% CI [0.68,0.75]

***Note.*** b represents unstandardized regression weights. *sr*^*2*^ represents the semi-partial correlation squared. A significant unstandardized regression coefficient (*b*) indicates the semi-partial correlation is also significant

**Figure 2:**
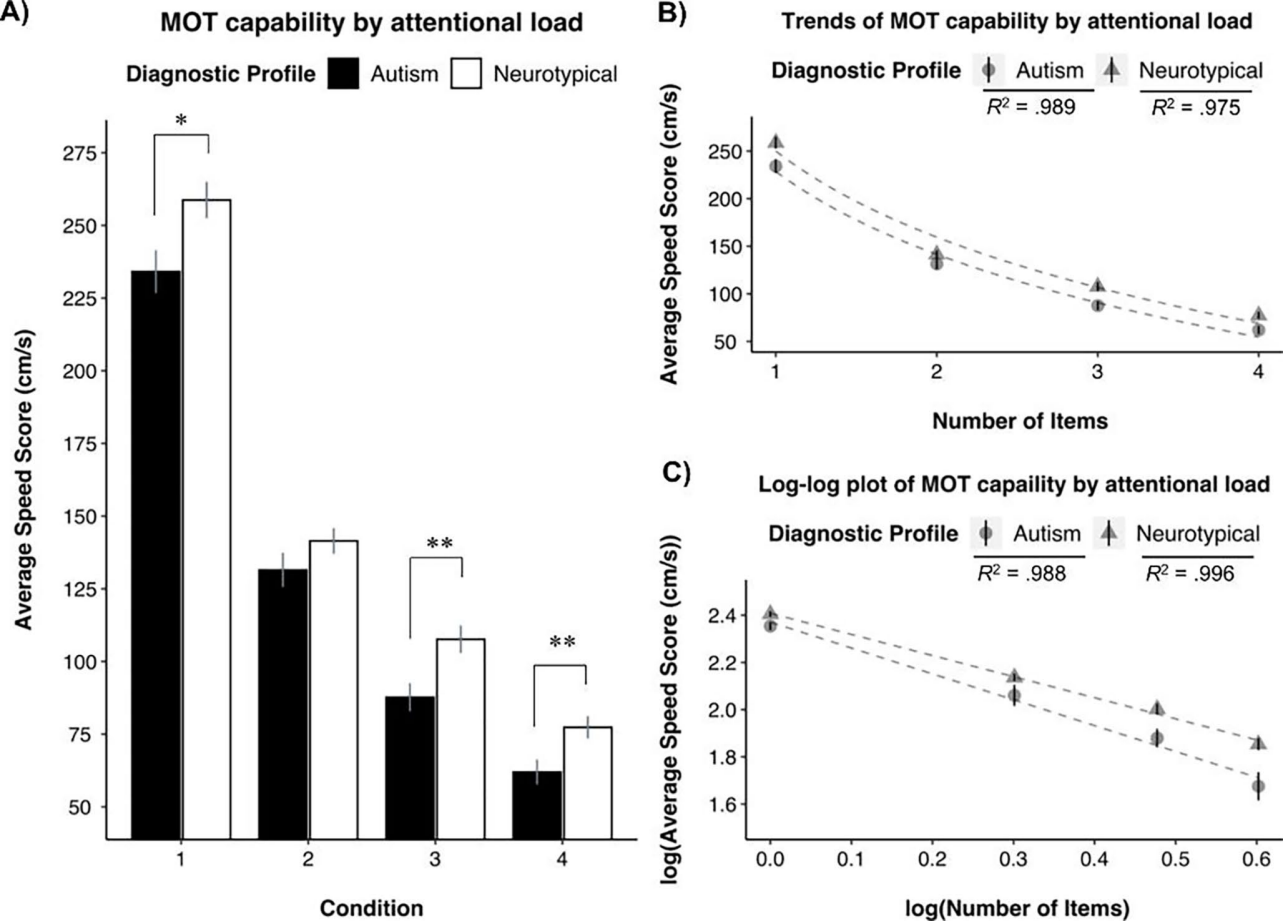
**(A)** Mean and standard error represented by bar plots across attentional load condition and between groups * *p* = .012; ** *p* = .004; *** *p* = .009. **(B)** MOT performance means and standard errors for each group plotted across condition on a linear plot. The decreasing logarithmic trends in performance are represented by the function *y* = -125.4ln(x) + 228.48 at *R*^2^ = .989 for the autism group; and y = -130.4ln(x) + 249.93 at *R*^2^ = .975 for the NT group. **(C)** The log of MOT performance plotted along the log of load condition for each group. The linear functions on the log-log plot are represented by y = -1.10x + 2.37 *R*^2^ = .988 for the autism group, and y = -0.89x + 2.41 at *R*^2^ = .996 for the NT group.

Four a priori independent samples *t*-tests were conducted to examine differences in MOT capability between the autism and NT group at each level of attentional load (see Fig. 2A). A statistically significant group difference suggesting that NT group performed better on MOT than the autism group was found when tracking one: *t*(108) = -2.55, *p* = .012, *d* = -0.49, 95% CI [-0.86, -0.11], three: *t*(108) = -2.95, *p* = .004, *d* = -0.57, 95% CI [-0.94, -0.18], and four target objects: *t*(108) = -2.67, *p* = .009, *d* = -0.51, 95% CI [-0.89, -0.13]; while no statistically significant difference was found for the two target object condition: *t*(108) = -1.36, *p* = .176, *d* = -0.26, 95% CI [-0.63, 0.12]). These results explain the non-statistically significant interaction in the first step of the hierarchical regression. Instead, the trends in MOT capability as attentional load increased between the two groups were parallel to one another, further suggesting no differences in the process of allocating attentional resources to task demands or ARC. To characterize these parallel trends between groups, we fit MOT capability at increasing levels of attentional load to a decreasing logarithmic function for both the autism (*y* = -125.4ln(x) + 228.48 at *R*^2^ = 0.989) and the NT groups (*y* = -130.4ln(x) + 249.93 at *R*^2^ = 0.975; see Fig. 2B). These trends replicate previous results in neurotypically developing adults (Alvarez & Franconeri, 2007; Tullo et al., 2020; Tullo, Faubert, et al. 2018).

Furthermore, we followed the protocol from previous research that examined the allocation of attentional resources to task demands using MOT by fitting a linear function to the log of the average speed scores plotted by the log of the number of target objects (see Alvarez and Franconeri 2007; Tullo, Faubert, et al. 2018). The linearity of performance plotted on the log-log plot suggests that the allocation of attentional resources to task demands are characterized as a proportion of object velocity and the inverse of object set size: y = *speed* * $$\raisebox{1ex}{$1$}\!\left/ \!\raisebox{-1ex}{$i$}\right.$$. A linear function best characterized the log-transformed data for both the autism (*y* = -1.10x + 2.37; at *R*^2^ = 0.988) and NT (*y* = -0.89x + 2.41; at *R*^2^ = 0.996) groups (Fig. 2C). Thus, the robust fit to linear trend on the log-log plot for both autism and NT groups provides more evidence that autistic and neurotypical participants’ performance on the MOT task as attentional load increased was decreased in parallel. In sum, there do not appear to be any differences in the way autistics and neurotypicals allocated attentional resources to task demands.

### Individual Differences Factors and MOT Capability

Next, we examined whether performance on a clinically validated measure of attention and intelligence (i.e., both verbal and fluid reasoning IQ as measured by the WASI-II VCI and PRI scores, respectively) explained the variance in MOT capability. The second step in the hierarchical regression yielded a significant model with attentional load level, group, the interaction between attentional load level and group, CPT-3 performance, fluid reasoning IQ (PRI), and verbal IQ (VCI) as predictors: *F*(6, 429) = 187.30, *p* < .001, *R*^2^ = .72, *Adj. R*^*2*^ = .72 (see Table 2). In the current model, attentional load: *b* = -57.80 *t*(429) = -23.08, *p* < .001, 95% CI [-62.72, -52.88]; and fluid reasoning IQ: *b* = 0.94 *t*(429) = -6.71, *p* < .001, 95% CI [0.66, 1.21]; were statistically significant predictors. The interaction between attentional load level and group remained a non-statistically significant predictor with the inclusion of CPT-3 and the two WASI-II intelligence subscales: *b* = 1.56 *t*(429) = 0.44, *p* = .660, 95% CI [-5.43, 8.56]. Furthermore, group: *b* = -12.61 *t*(429) = -1.27, *p* < .001, 95% CI [-32.15, 6.92], CPT-3 performance (i.e., d’ t-score): *b* = -0.33, *t*(429) = -1.59, *p* < .001, 95% CI [-0.73, 0.08], and verbal intelligence: *b* = -0.15, *t*(429) = -1.11, *p* < .001, 95% CI [-0.41, 0.11] were not statistically significant predictors. The model with CPT-3, fluid reasoning (PRI), and verbal (VCI) IQ as additional predictors resulted in a statistically significant contribution to the variance in MOT performance compared to the regression model in step 1: Δ*F*(3, 429) = 24.99, *p* < .001, Δ*R*^2^ = 0.05, Δ*Adj. R*^2^ = 0.05. These results, therefore, suggest that fluid reasoning intelligence, as defined by the WASI-II PRI score, accounts for a significantly large portion of the variance in MOT capability compared to either performance on the CPT-3 or verbal intelligence (VCI score). Moreover, when accounting for fluid reasoning intelligence the group effect was no longer statistically significant, suggesting that MOT performance is associated with higher-order cognition but not the diagnostic characteristics of the autistic phenotype. In addition, these results suggest that MOT capability can further characterize attentional capability beyond traditional and currently accepted clinical measures since CPT-3 performance (i.e., a traditional and clinically validated measure of attention) did not predict ARC.

### Intellectual Style and MOT Capability

Lastly, we examined differences in intellectual styles both between and within autism and NT groups. Using a procedure similar to Gevins and Smith (2000), participants’ intellectual styles were categorized into verbal and fluid reasoning IQ categories, where fluid reasoning IQ (i.e., PRI scores on the WASI-II) were subtracted by verbal IQ (i.e., VCI scores on the WASI-II), and then divided by global IQ scores (i.e., FSIQ scores on the WASI-II). Using this discrepancy score, those equal to or above the 75th percentile rank relative to the participants’ group were defined as a “fluid reasoning intellectual style”, and those equal to or below the 25th percentile rank were defined as a “verbal intellectual style”. This procedure resulted in an equal distribution of participants defined by intellectual style by group, with 14 participants in each cell (see Table 3).

**Table 3:** *Means and variances for WASI-II and MOT between intellectual styles and group*

Group	Intellectual Styles	FSIQ	VCI	PRI	
**Neurotypical**	Verbal (n = 14)	104.14 (16.19)	111.43 (15.06)	94.86 (13.07)	
**Neurotypical**	Fluid Reasoning (n = 14)	103.36 (12.97)	92.43 (11.51)	115.14 (13.11)	
**Autism**	Verbal (n = 14)	92.36 (28.76)	101.50 (19.94)	83.93 (24.70)	
**Autism**	Fluid Reasoning (n = 14)	90.64 (17.91)	78.50 (15.32)	105.71 (19.94)	

***Note.*** Means and (standard deviations) reported for 14 participants in both verbal and fluid reasoning intellectual styles

Given the discrepancy in fluid reasoning intelligence over verbal intelligence, characteristic of the autistic population (see Dawson et al. 2007; Nader et al. 2015), we compared the discrepancy between fluid reasoning and verbal intelligence scores between autism and NT groups using an independent samples *t*-test. The results revealed no differences in discrepancy scores between autistic (*M* = 0.06, *SD* = 0.20) and NT groups (*M* = 0.03, *SD* = 0.15): *t*(107) = -0.87, *p* = .388, *d* = -0.17, 95% CI[-0.54, 0.21], suggesting fair comparisons between intellectual styles, both between and within groups.

To assess whether MOT capability differed across intellectual styles between and within groups, we conducted a three-way mixed-design ANOVA with group and intellectual style as between-subjects factors, and attentional load condition as the within-subjects factor. With the trimmed sample representing participants with a significant discrepancy in verbal and fluid reasoning IQ scores, there was no statistically significant interaction between group and style *F*(1, 52) = 0.28, *p* = .600, partial η^2^ = 0.01, 95% CI [0.00, 0.11] nor a statistically significant interaction between load and group: *F*(1, 52) = 0.38, *p* = .538, partial η^2^ = 0.01, 95% CI [0.00, 0.12]. Similarly, there was no statistically significant interaction between intellectual style and load condition: *F*(1, 52) = 0.00, *p* = .957, partial η^2^ = 0.00, 95% CI [0.00, 0.02], as well as no statistically significant three-way interaction between group, style and load condition: *F*(1, 52) = 0.08, *p* = .778, partial η^2^ = 0.00, 95% CI [0.00, 0.08].

Furthermore, the results demonstrated a statistically significant main effect of load condition (*F*(1, 52) = 831.72, *p* < .001, partial η^2^ = 0.94, 95% CI [0.90, 0.96]). Yet, there was no statistically significant main effect of diagnosis (*F*(1, 52) = 1.05, partial η^2^ = 0.02, 95% CI [0.00, 0.15]). Additionally, the analysis revealed a statistically significant main effect of intellectual style: *F*(1, 52) = 11.79, *p* = .001, partial η^2^ = 0.13, 95% CI [0.03, 0.39]. In sum, the results demonstrated that tracking capability differed between intellectual styles, where individuals with a fluid reasoning intellectual style showed greater MOT tracking capability; however, there was no effect nor interaction with group. These results provide additional evidence suggesting a strong and robust link between ARC, as measured by MOT capability, and fluid reasoning intelligence.

## Discussion

In this study, we sought to characterize attention resource capacity in autistic persons using an MOT paradigm. We measured MOT performance by calculating the maximum speed at which participants accurately tracked all target items as the attentional load increased (Meyerhoff et al., 2017; Scholl, 2009). Our results showed that neurotypical individuals outperformed autistics in MOT capability across different levels of attentional load, but this difference was explained by differences in Wechsler-defined fluid reasoning intelligence. However, we found no significant differences in how autistic and neurotypical individuals allocate attentional resources to task demands, as both groups showed similar logarithmic decreases in MOT performance as the attentional load increased. We also found that individuals with a fluid reasoning intellectual style demonstrated greater MOT capability compared to those with a verbal intellectual style, regardless of group. Taken together, these findings extend previous research conducted in neurotypical adults to autistics (Tullo et al., 2020; Tullo, Faubert, et al. 2018). Also, these results have theoretical implications for understanding attentional capability when capacity limits are reached, and practical implications for clinicians assessing attention in autistics with varying cognitive abilities.

Previous research that examined MOT capability in autism, such as studies that isolated visuoperceptual capacity limits (see Evers et al. 2014; Koldewyn et al. 2013; Van der Hallen et al. 2015), focused on examining the mechanisms involved in visual tracking rather than using MOT as a measure of attentional capability (Tullo, Faubert, et al. 2018). For example, some studies have examined specific sub-components of attention as measured by MOT by grouping target and distractor objects, defining performance by an object limit, and focusing on the underlying mechanisms involved in visual tracking (Evers et al., 2014; Koldewyn et al., 2013; Van der Hallen et al., 2015). This approach to examining MOT capability in autism has similar limitations to research on characterizing MOT capability in neurotypicals, where a consensus has not been reached regarding how to determine an object limit that defines attention capacity (Suchow et al., 2014; Tullo, Faubert, et al. 2018). Previous research using MOT paradigms in autism has found decreased (Evers et al., 2014; Koldewyn et al., 2013) and equal MOT performance relative to neurotypically-developing participants (Van der Hallen et al., 2015). The current study borrowed the approach used to examine attention resource capacity in neurotypically developed adults where a continuous ratio variable (i.e., average speed score) provided the opportunity to: (i) isolate and capture attentional resources allocated to task demands and (ii) evaluate variables that can account for individual differences in MOT performance, such as higher-order cognition (Tullo et al., 2020; Tullo, Faubert, et al. 2018). This approach has allowed us (i) to demonstrate that autistics and neurotypicals allocate attentional resources to task demands in a similar manner, and (ii) afforded the opportunity to assess and demonstrate that attention resource capacity is best accounted for by fluid reasoning intelligence. Thus, the evidence accrued throughout this endeavor further advocates for the use of speed as an appropriate characterization of MOT capability (Chen et al., 2013; Holcombe & Chen, 2012, 2013; Tombu & Seiffert, 2008) and presents a more ecologically valid characterization of attention (Tullo, Faubert, et al. 2018).

Previous research has examined the allocation of attentional resources to task demands in neurotypical adults by assessing MOT performance at increasing attentional load, and this decreasing logarithmic trend has been consistently observed (Alvarez & Franconeri, 2007; Tullo et al., 2020; Tullo, Faubert, et al. 2018). The results presented in the current study replicate and extend this trend to autistics across a broad range of ages and intelligence. Specifically, we highlight the similarity between autistics and neurotypicals as evidenced by the fit of MOT performance with increasing attentional load to the decreasing logarithmic trend. This similarity between groups depicts the allocation of attentional resources to task demands, where these limited resources are allocated to object speed and set size (i.e., the number of target objects), equally. This similarity in the allocation of attentional resources between groups, in addition to the absence of an interaction effect of attentional load by group, suggests a clear distinction between attentional and perceptual capabilities in autism.

Perceptual load theory suggests that when available resources are allocated to distractors, they can be processed in conditions of low perceptual load, while they are not processed in conditions of high load due to the priority assigned to target stimuli (Lavie, 2005). Moreover, previous research has shown that autistics have superior perceptual capacities compared to neurotypicals given that can incorporate distractors at high levels of perceptual load (Remington et al., 2009, 2012); yet, allocating attentional resources to distractors may hurt task performance (Adams & Jarrold, 2012). However, our study did not find differences in MOT performance, our proxy for attention resource capacity, between autistics and neurotypicals. Instead, our results suggest that MOT performance varied as a function of higher-order cognition, defined by fluid reasoning intelligence. Our findings, therefore, suggest that attention resource capacity may represent distinct mechanisms and processes from perceptual capacity.

The separation between perceptual and attentional constructs could be explained by the multi-dimensional nature of attention compared to perceptual load (Heitz et al., 2005; Lavie, 1995). Attention resource capacity, as measured by the MOT paradigm, is comprised of distributed, sustained, and selective sub-components of attention. In the current study, MOT performance was defined as the maximum speed participants could distribute and sustain attentional resources on target items throughout the tracking phase, while selectively suppressing the allocation of resources to distractor objects. Here, the allocation of attentional resources to task demands was similar for autistics and neurotypicals, as depicted by the linearity of maximum speed by load condition on the log-log plot for both groups (see Fig. 2C). Additionally, the distinction between perceptual and attentional load can be explained by the difference in the effect and role of distractors in MOT compared to other perceptual tasks (Remington et al., 2009, 2012). The role of processing distractors in selective attention when assessing perceptual load theory in autism does not result in a failure to complete the task; instead, processing distractors prolongs the time to complete the visual search task (Remington et al., 2009, 2012). Whereas, incorrectly allocating resources to distractor items in MOT results in dropping target items, which in turn, results in task failure (Drew et al., 2013). As such, perceptual capacity and attentional capacity are separate constructs. Therefore, the inconsistent results characterizing MOT capability in autism can be attributed to the use of MOT as a metric of visuoperceptual capacity (i.e., defining performance as an object limit; Evers et al. 2014; Van der Hallen et al. 2015) rather than as an assessment of attentional capability (i.e., defining performance as attentional capacity; Tullo et al. 2020; Tullo, Faubert, et al. 2018).

The results from the current study also demonstrated (i) a significant positive relationship between attention resource capacity and fluid reasoning intelligence, and (ii) increased tracking capabilities for individuals with a fluid reasoning intellectual style compared to a verbal intellectual style. As such, the association between fluid reasoning intelligence and attention resource capacity adds to the knowledge of attention in autism and extends beyond theoretical implications to practical implications. These findings advocate for the need to contextualize attentional capability in autism with higher-order cognition, such as fluid reasoning intelligence. Clinical assessments of attention are limited to performance on behavioral measures of attention (i.e., CPT-3) and subjective informant-based ratings using questionnaires (Hulme & Snowling, 2013); thus, these assessments may not account for higher-order cognition, nor provide an ecologically valid assessment of the individual’s capability to allocate attention to task demands (Powell et al., 2017; Scerif, 2010). This limitation challenges the accuracy of the assessment because impairments in attention-related domains that are characteristic of the ASD phenotype are related to higher-order cognitive functioning (Ben-Itzchak et al., 2014). For instance, autistics exhibit atypical joint-attention (Frith et al., 2003; Mundy & Sigman, 1989; Van Hecke et al., 2016) and pay less attention to socially salient information (e.g., facial expressions and hand gestures; Elsabbagh et al. 2014). Therefore, the addition of fluid reasoning intelligence can add further context to the individual’s attentional capability as a function of their cognitive competency (Paas & van Merriënboer, 1994; Tullo, Faubert, et al. 2018) and the expression of behavioral characteristics specific to ASD (Ben-Itzchak et al., 2014). Future research exploring the link between attentional capabilities and ASD phenotype could examine the relationship between deploying attentional resources to task demands, specific to measures of social attention.

## Limitations

While our study provides valuable insights on characterizing attention resource capacity in autism as a function of higher-order cognition, there are limitations that should be considered when interpreting the findings and for future research to advance the knowledge on the topic. The considerations taken in participant recruitment to isolate autistic phenotype may impact the generalizability of the findings.

For one, we excluded participants with a deficit in attention and matched performance on a clinically validated measure of attention with the neurotypical group. We recognize that this decision may limit the generalizability of our findings to the broader autistic population, given the high comorbidity between ADHD and ASD (Davis & Kollins, 2012; Leitner, 2014; Sikora et al., 2012). In fact, these inclusionary criteria and matching based on age and attention may have caused the imbalance in sex and intelligence between the two groups of interest. Yet, this exclusionary criterion afforded us the opportunity to isolate the autistic phenotype.

Another limitation of the study is that the autistic group sample was recruited from a hospital and a school that provides specialized learning programs for individuals with alternative learning preferences, and did not include community members (cf., Participants). As such, this might explain the imbalance in intelligence scores compared to the neurotypical sample. Future research will be well served to expand recruitment to include participants recruited throughout the community to improve the generalizability of the findings.

Lastly, the lack of statistical power to interpret the interaction between intellectual style and the diagnostic group is another limitation. As intellectual styles are naturally occurring groups, it is challenging to account for statistical power to answer this research question. Nevertheless, our sample size is comparable to findings from previous work that found an effect of intellectual style (Tullo, Faubert, et al. 2018). Additionally, we found no differences in the degree of bias between fluid reasoning and verbal scores on the WASI-II across autistic and neurotypically developing groups. While these results may have been influenced by participant recruitment of the autistic group, limited subtests offered by the abbreviated measure of intelligence, and out of the scope of the current study, the data does not suggest a superiority in non-verbal intelligence over verbal intelligence measures for autistics.

We equally acknowledge the significant range of the autistic participant’s intelligence included in the present study is novel and is a better representation of the heterogeneity in the autism spectrum compared to research with strict inclusionary criteria. Although previous research examining MOT capability in autism uses intelligence measures as an inclusionary criterion (Evers et al., 2014; Koldewyn et al., 2013; Van der Hallen et al., 2015), the present study is the first to characterize attention resource capacity while accounting for intelligence. Therefore, the results from the current study encourage future research to further characterize individual differences in attention resource capacity with factors that can account for the heterogeneity in autism, such as those reflecting higher-order cognition (i.e., level of functioning, type of autism, etc. Ben-Itzchak et al. 2014) and intellectual style (Gevins & Smith, 2000; Tullo, Faubert, et al. 2018).

## Conclusion

The current study investigated whether allocating attentional resources to task demands and the size of the limited capacities of these attentional resources differed as a function of autism phenotype, cognitive capability, or both. We assessed these objectives by repurposing the MOT task as an ecologically valid measure of attention. Additionally, we exploited the versatility of the task by manipulating the attentional load (i.e., the number of target objects relative to distractors) affording the opportunity to characterize the allocation of resources to task demands and explore differences in attention resource capacity between and within autism and neurotypical groups. As a result, there is no evidence to suggest differences in the allocation of attentional resources nor the size of the limited capacities between autistics and neurotypicals. Instead, attentional capability varies as a function of higher-order cognition, more specifically, fluid reasoning intelligence. Overall, the results from the current study distinguish perceptual load and attentional load and further advocate for the use of MOT as a descriptor of attention and cognitive competency. Future research can benefit from the measure’s sensitivity to capture individual differences representing the heterogenous autistic population to further characterize attention and autism.

## Abbreviations

MOT: Multiple Object Tracking
ASD: Autism Spectrum Disorder

## Footnotes

1 We use ‘autism’ and ‘Autism Spectrum Disorder’ interchangeably to refer to the diagnosis of Autism Spectrum Disorders, and we predominantly use identity-first language (e.g. ‘autistic person’) rather than person-first language (e.g. ‘individual with autism’), in order to respect the terminology preferences of the majority of the autistic community (see Bury et al. (2020) and Kenny et al. (2016).
